# Identification of cis-regulatory elements associated with salinity and drought stress tolerance in rice from co-expressed gene interaction networks

**DOI:** 10.6026/97320630014123

**Published:** 2018-03-31

**Authors:** Pragya Mishra, Nisha Singh, Ajay Jain, Neha Jain, Vagish Mishra, Pushplatha G, Kiran P. Sandhya, Nagendra Kumar Singh, Vandna Rai

**Affiliations:** 1 National Research Centre on Plant Biotechnology, Indian Agriculture Research Institute, New Delhi, India; 2 Banasthali University, Tonk, Rajasthan; 3 Ocimum Biosolutions, Hyderabad, India

**Keywords:** cis-regulatory elements, drought stress, gene interaction network, microarray, rice, salt stress

## Abstract

Rice, a staple food crop, is often subjected to drought and salinity stresses thereby limiting its yield potential. Since there is a cross talk between these abiotic stresses, identification of common and/or overlapping regulatory elements is pivotal for generating rice cultivars that showed tolerance towards them. Analysis of the gene interaction network (GIN) facilitates identifying the role of individual genes and their interactions with others that constitute important molecular determinants in sensing and signaling cascade governing drought and/or salinity stresses. Identification of the various cis-regulatory elements of the genes constituting GIN is equally important. Here, in this study graphical Gaussian model (GGM) was used for generating GIN for an array of genes that were differentially regulated during salinity and/or drought stresses to contrasting rice cultivars (salt-tolerant [CSR11], salt-sensitive [VSR156], drought-tolerant [Vandana], drought-sensitive [IR64]). Whole genome transcriptom profiling by using microarray were employed in this study. Markov Chain completed co-expression analyses of differentially expressed genes using Dynamic Bayesian Network, Probabilistic Boolean Network and Steady State Analysis. A compact GIN was identified for commonly co-expressed genes during salinity and drought stresses with three major hubs constituted by Myb2 transcription factor (TF), phosphoglycerate kinase and heat shock protein (Hsp). The analysis suggested a pivotal role of these genes in salinity and/or drought stress responses. Further, analysis of cis-regulatory elements (CREs) of commonly differentially expressed genes during salinity and drought stresses revealed the presence of 20 different motifs.

## Background

Rice (Oryza sativa) is one of the major food crops for more than 3.5 billion population of the world. It was estimated that for an additional 1 billion population, 100 million additional tons of rice would be required but with limited resources. Rice is largely grown under rain fed condition [1] and often subjected to various types of abiotic stresses such as salinity [2, 3] and drought [4]. In India, 9.04 million hectare of rice growing area is affected by salinity leading to considerable loss of grain yield. Not only salinity stress but frequently occurred severe droughts also drastically affect rice production [5]. Salinity and drought stresses causes osmotic imbalance on plants. There are several studies that have shown extensive cross talk between different abiotic stresses. Therefore, it is logical to assume a common regulatory mechanism(s) that may exert influence on sensing and signaling cascade governing these abiotic stresses. Some creditability towards this assumption could be gained from studies where manipulation of a single molecular determinant could confer tolerance towards two different abiotic stresses [6]. Comparative transcriptomic profiling has been employed for identification of genes related to abiotic stress tolerance. DNA microarrays are one of the techniques to identify stress related genes for multiple stress tolerance [7]. In rice cDNA libraries, expressed sequence tags (ESTs) and microarray data were generated for salt tolerant and salt sensitive varieties to examine changes in roots of control and salt stressed plants [8]. Affymetrix rice gene chips were used to study the expression of genes in FL478 (recombinant inbred line) as salt tolerant and IR29 as salt sensitive rice [9].

Construction of co-expression gene interaction networks (GINs) can be one of the approaches to study changes within two conditions and thus deciphering underlying biochemical pathways. Differential network analysis in conjunction with gene expression data provides a deeper understanding than that of just list of DEGs and can be a reflection of functional association between two genes [10]. Gene network is a process to identify gene interactions from experimental data sets through computational analysis and microarrays were normally employed [11]. A co-expression network was created for rice using Affymetrix microarray data [12]. Thus, a co-expression gene interaction network (GIN) analysis can help in elucidating the complex interactions across several genes that helps in defining consequent cascade of biochemical events from these interactions. By integration of co-expression data together with promoter analysis, cis-regulatory elements were identified to study cross talk of salinity and senescence induced signal transduction [13]. Transcription factors and cis-elements of stress responsive promoters function as molecular switches as well as end point for signaling machinery [14]. Members of AP2, bZIP, MYB, zinc-finger TFs were stress responsive regulators [15]. Over-expression of OsDREB1A, OsDREB1B, OsNAC6/SNAC2 improved abiotic stress tolerance (cold, salinity and drought) of rice transcriptional regulation of Cytochrome P450 (CYP) 735A in regulating cytokinin level under cold and dehydration stress [16]. However, the common regulatory elements governing salinity and drought stress responses are far from being elucidated. In the present study, co-expression gene interaction networks were identified for differentially expressed genes in salinity and drought stresses along with cis-regulatory elements to study sensing and signaling mechanisms evolved through cross talk. The results provide novel insights towards the common regulatory mechanisms responsive to salinity and drought stress tolerance.

## Methodology

### Experimental Design

Each experimental condition consists of two rice varieties, tested under non-stressed (control) and stressed (experimental) conditions. The experiment was aimed at studying the expression levels of genes under salt and drought stress in salt tolerant CSR11, salt-sensitive-VSR156 and drought-tolerant Vandana and drought-sensitive IR64. For salt stress seeds of CSR11 and VSR156 were sown with Hoagland's nutrient solutions and after 14 days transferred to control and 150mM NaCl containing media. After 24h leaf samples were collected for RNA extraction. For drought stress seeds of Vandana and IR64 were sown in Hoaglands nutrient solution. After 14 days seedlings were blot dried and kept in air for 12 hrs and leaf samples were collected for RNA extraction.

### RNA isolation and Gene Chip hybridization

The seeds of CSR11 (salt tolerant), VSR156 (salt-sensitive), Vandana (drought-tolerant), IR64 (drought-sensitive) were sown in magenta boxes in replicates using standard growth conditions. Ten days after germination, 150mM NaCl was added to the media for imparting salinity stress in one set while another set was grown as control without NaCl. For drought stress 10 days after germination seedlings of Vandana and IR64 were kept outside (without media) for 12h and one set was kept in normal condition for control. After 24 h of salt stress and 12 h of drought stress shoot sample were collected washed and pooled in equal amounts for RNA extraction using TRIzol Reagent (Sigma). Plant tissues were homogenized using mortar and pestle with liquid nitrogen and 1 ml of TRIzol reagent was added per 100 mg of tissue. RNA samples were processed according to Affymetrix Gene Chip expression analysis technical manual. The cDNA was synthesized from poly (A)+ mRNA present in 8 μg of total RNA using Superscript double-stranded cDNA synthesis kit and poly (T) nucleotide primers that contain sequence recognized by T7 RNA polymerase. A portion of the resulting double stranded cDNA was used as template to generate biotin-tagged cRNA from an in vitro transcription reaction, using Affymetrix Gene Chip IVT labeling kit. Fifteen micrograms of biotin tagged cRNA were segmented to strands of 35 to 200 bases length using Affymetrix protocols. Subsequently, 10 μg of this fragmented cRNA was hybridized at 45°C with rotation for 16 h in Affymetrix Gene Chip hybridization oven 450 to the Affymetrix rice Gene Chip arrays. The Gene Chip arrays were washed and then stained with streptavidin-phycoerythrin in Affymetrix Fluidics station 450, followed by scanning on a Gene Chip Scanner 3000.

### Rice Gene Chip array and probe annotation

The Affymetrix rice genome array contained probe sets designed from 48564 japonica and 10260 indica gene sequences. The sequence information for this array was derived from the National Center for Biotechnology Information (NCBI) UniGene build number 52 (http://www.ncbi.nlm.nih.gov/UniGene), GenBank mRNAs, and 59,712 gene predictions from TIGR's osa1, version 2.0. Gene models that had any indication of transposable elements were removed from the list of TIGR genes. The array is believed to represent about 46,000 distinct rice genes. About 26,000 of these are 3' anchored Unigene ESTs and mRNA clusters, including known rice full-length cDNA clones, and 19,431 are solely from the TIGR gene predictions. Microarray data from this study have been deposited in NCBI database at Gene Expression Omnibus with the accession number GSE1651. To obtain annotations for the salt-regulated probe sets, we extracted the target sequence of identified probe sets from the sequence information file (.sif) for the rice genome array. The target sequence extends from the 5'- end of the 5-most probe to the 3'- end of the 3-most probe. The target sequences were then searched using BLASTN software against the TIGR rice pseudomolecules, release 6 (http://rice.plantbiology.msu.edu). The microarray data had submitted to GEO and accession number is GSE21651.

### Validation of differentially expressed genes for common GIN (genes which are common to drought and salt stress) by quantitative real time PCR

Expression levels of 10 genes (Additional file 8: Table S8) were analyzed using real-time, quantitative PCR for the validation of microarray results. The sequence of each gene was downloaded from TIGR rice database (http://rice.plantbiology.msu.edu). Exonic sequences were used for design of primers using Beacon Designer™. We used eEF-1a (eukaryotic elongation factor 1- alpha) as an internal control for consistent results. Quantitative real-time PCR (qRT-PCR) was conducted according to protocol described by the Invitrogen (Catalog no- 11736-051) using the SuperScript III Platinum SYBER Green One-Step qRT-PCR Kit. Thermal cycling conditions comprised of 50°C for 1 h followed by an initial denaturation at 95°C for 10 min, followed by 40 cycles of denaturation at 95°C for 30 sec, annealing at 55-65°C for 1 min and extension at 72°C for 1 min. All qRT-PCR reactions were performed in the Stratagene Mx3000p followed by analysis of dissociation curve, taking a fluorescence reading at every degree between 55°C and 95°C to ensure that only one PCR product was amplified. The experiments were performed in six replicate means sampling was done from two independent experiments, for each data point and normalized against eEF-1a amplification to ensure that the differential expression was not due to differing amount of initial RNA template. The data analysis was performed using Mxpro-QPCR software (Stratagene).

### Statistical analysis of micro array data

The array data set was analyzed using Gene Chip Operating Software (GCOS 1.2) and Genowiz software. Probe level normalization (RMA) was performed on Affymetrix raw files (CEL files). Initial dataset consisted of 57,382 probes. Filtration was performed to remove probes with 'Absent Calls' (Poor quality probes). Transformation and normalization was done in order to facilitate comparison across samples. To determine biological significance of differentially expressed genes, functional classification was performed using Gene Ontology. Gene Ontology reports along with z score. Functional classification was performed using BLASTX search (http://blast.ncbi.nlm.nih.gov/Blast).

### Data Normalization

CEL files obtained for each sample were processed through RMA algorithm, which consists of probe level background correction, normalization and probe set summarization. In order to detect if the expression data for any sample has any abnormalities, intensity distribution of samples before and after summarization was studied through Box plots (not shown here). The pair wise correlation between the expression values for samples was studied through Pearson's correlation coefficient. The analysis resulted into a correlation matrix indicating the extent of linear relationship amongst samples. A coefficient value close to 1.0 indicates linear relation between the two arrays.

### Differentially Expressed Genes

The normalized expression data on samples after removal of the control probes was used to identify probe sets that are differentially regulated across the comparisons of interest. Twosample t-test with Welch's correction for degrees of freedom had used to determine differentially expressed genes. A threshold pvalue of 0.05, and the log fold-change threshold was set to 1. These settings were retained throughout the analysis to select gene list across different comparisons.

### Determination of Co-Expressed Genes and Gene Network Analysis

Post Expression data analysis for identifying co-expressed genes, as construction of networks is the 'Gene Interaction Network', which is based on principles like 'Dynamic Bayesian Network'. Bayesian networks [17] are directed acyclic graphs whose nodes represent variables, and whose missing edges encode conditional independencies between the variables. The data after standardization was processed for determination of co-expressed genes. Based on experimental design contrasts are defined to compare samples across groups thereby identifying probe-sets that are differentially expressed. For each probe-set a linear model is built for the contrast of interest using Robust Linear Model (RML) method and p-values are generated. Since the analysis involves large number of tests, one for each probe-set, pvalues are adjusted using Benjamin and Hochberg's FDR correction. Probe-sets that are differentially expressed in each comparison, at a p-value threshold of 0.05, are mapped to their corresponding genes and these genes were further studied to identify critical molecular functions and pathways affected by differential gene signatures. The Up and Down regulation of signatures are analyzed to determine the Gene Interaction Networks for the salt and drought treatment specific factors. The correlation networks were identified by two simple steps: i) the computation of all pairwise correlations for the investigated variables, and ii) a threshold or filtering procedure to identify significant correlations, and hence edges, of the network. However, for shedding light on the causal processes underlying the observed data, correlation networks are only of limited use. This is due to the fact that correlations not only confound direct and indirect associations but also provide no means to distinguish between response variables and covariates (and thus between cause and effect). Therefore, causal analysis requires tools different from correlation networks: much of the work in this area has focused on Bayesian networks or related regression models such as systems of recursive equations or influence diagrams. All of these models have in common that they describe causal relations by an underlying directed acyclic graph (DAG). The differentially expressed probe sets obtained in each comparison were used to build Gene Interaction Networks using Gene Net and R graph viz., packages of R. Determination of Network proceeds in two steps as follows:

First, the correlation network was transformed into a partial correlation network, which was essentially an undirected graph that displays the direct linear associations only. This type of network model is also known under the names of graphical Gaussian model (GGM) [18] concentration graph, employed in Arabidopsis wherein a network of 18,625 interactions (edged) for 6760 genes (nodes) were identified [19].

The undirected GGM was converted into a partially directed graph by estimating a pair-wise ordering of the nodes from the data using multiple testing of the log-ratios of standardized partial variances, and by subsequent projection of this partial ordering onto the GGM. The inferred causal network is the sub graph containing all the directed edges. Heuristic algorithm for discovering approximate casual networks was used to obtain Gene Interaction Network.

Co-expression analysis of genes was done with 'Dynamic Bayesian Network', 'Probabilistic Boolean Network', and 'Steady State Analysis by Markov Chain'. Bayesian networks are directed acyclic graphs whose nodes represent variables, and whose missing edges encode conditional independencies between the variables. Let G = (V, E) be a directed acyclic graph (or DAG), and let X = (Xv)v _ V be a set of random variables indexed by V. X is a Bayesian network with respect to G if its joint probability density function can be written as a product of the individual density functions, conditional on their parent variables: where pa(v) is the set of parents of v [20].

Probe-sets that are found to be significantly differentially expressed in each comparison, at a p-value threshold of 0.05, are mapped to their corresponding genes and these genes are further studied to identify critical molecular functions and pathways affected by differential gene signatures. The Up and Down regulation of signatures are analyzed to determine the Gene Interaction Networks for the treatment specific factors. The differentially expressed probe sets obtained in each comparison were used to build Gene Interaction Networks using Gene Net and R graph viz., packages of R. The correlation networks were constructed as graphical Gaussian model (GGM) [18] Heuristic algorithm for discovering approximate casual networks is used to obtain Gene Interaction Network.

### Gene Enrichment Analysis

The genes obtained through various comparisons were studied for their over-abundance in different GO terms as well as Pathways. The terms could be categorized into biological process, molecular function and cellular component. The over-abundance of a particular term could be decided based on the number of significant genes in the analysis, the number of significant genes relevant to the term, the total number of genes for the organism and the number of genes that are relevant to the term for the organism. Fisher's exact test could be used to determine the significance of the GO term. If a term is significant at p< 0.05, then it is implied as enriched with genes. Accordingly, the biological relevance of the term and the associated genes could be explored. Similar description holds for pathways analysis. In the present study, for GO analysis, the data from Gene Ontology consortium was used, while for pathways, human KEGG pathways were referred.

### Transcription Factor Analysis

In plants, a large number of transcription factors (TF) are known to control the expression of target genes in various signal transduction cascades. Thus, the 1000 bp upstream sequences i.e. the promoter regions of the common network genes in salt and drought types were scanned for the transcription factor binding sites. Osiris tool was used to found out the transcription factor binding sites in the common genes and were depicted by number of promoters bound in the subset, promoters in the genome, pvalue and the corresponding motif sequence for each of the site. Each of the motifs was further analyzed for the position, strand and location in the genomic sequences.

### Motif analysis

The "MSU locus" sequences were downloaded from the "MSU Rice Genome Annotation Project". Multi fasta file representing all loci in the respective tables were split into part files with the character limit of 60,000 maximum in each part file, due to the limitation of the motif prediction tool. All the motifs in the sequences were predicted using the MEME (Multiple EM for Motif Elicitation) tool on forward as well as reverse strand and contain no gaps [21]. The overall height of each stack indicates the sequence conservation at that position (measured in bits), whereas the height of symbols within the stack reflects the relative frequencies of the corresponding nucleic acid at that position.

MEME represents motifs as position-dependent letter-probability matrices, which describe the probability of each possible letter at each position in the pattern. For parameter setting we opted for ZOOPS (Zero or one per sequences) distribution of motifs across the sequences and motifs length within range 6-15. The total number of sites opted for the training set was minimum 2 and maximum number was equivalent to the "number of the sequences in the multi-fasta file or part file". The predicted distinct motifs with similarity <60% by MEME are listed under section "Significant motifs" in each spreadsheet. The predicted motif was searched against the sequence database for the occurrences using MAST (Motif-based sequenced analysis tool). MAST assumes exactly one occurrence of each motif per sequence, and each sequence in the database is assigned a pvalue, based on the product of the p-values of the individual motif occurrences in that sequence. The motifs predicted were compared and validated against the "JASPAR_CORE_2014_plants database" using TOMTOM tool. Total 64 motifs were read from this database after removing those, which has conflicting Ids. The significant matches were listed against each motif in the section "Significant motifs".

## Results and discussion

### Differentially expressed genes (DEG) between tolerant and susceptible varieties under salt and drought stress

**Figure 1:**
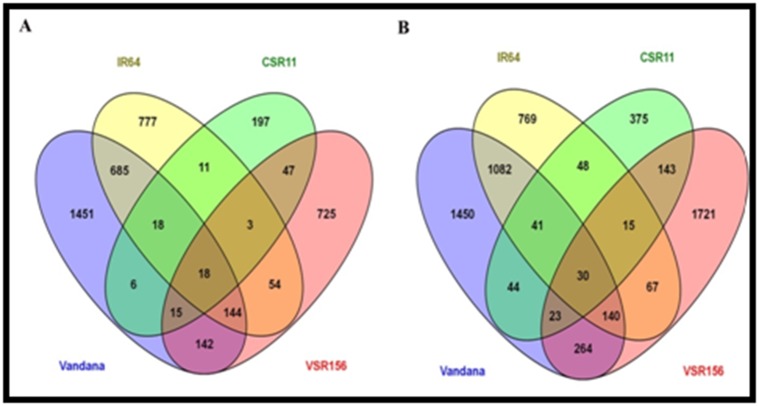
Number of genes differentially expressed during salinity and drought stress responses in contrasting genotypes. Rice seedlings (14-day-old) of salt-sensitive (SS) VSR156, salt-tolerant (ST) CSR11, drought-sensitive (DS) IR64 and drought-tolerant (DT) Vandana genotypes were grown hydroponically under controlled growth conditions and subjected to salinity (NaCl 150 mM) and drought stress treatments for 24 h and 12 h, respectively. Corresponding genotypes grown under normal condition were used as respective controls. Whole plants (control and treated) were used for global microarray analysis using Affymetrix gene chip. Venn diagram represents number of genes that were differentially expressed under salinity and/or drought stress conditions.

Salt tolerant (CSR11) and salt sensitive (VSR156) rice varieties were grown under normal growth conditions and 14-day-old seedlings were exposed to salt stress (150mM NaCl) for 24 hrs. In addition, drought tolerant (Vandana) and drought sensitive (IR64) rice varieties were grown under control (irrigated) and 14- day-old seedlings were exposed to drought stress (12 hrs) conditions. The control and stressed seedling were then used for microarray gene expression profiling using Affymetrix system. A total of 315 and 720 gene probes were up and down regulated in CSR11 under salt stress conditions compared to the control seedlings. Relatively, salt-stressed VSR156 compared to the control revealed significantly higher number of gene probes that showed differential regulation (induction of 1148 gene probes and suppression of 2403 gene probes (Figure 1). Comparative transcriptomics was done using DNA microarrays in rice for salttolerant (Pokkali) and salt-sensitive (IR29) varieties and 10% genes were differentially expressed within 1 hr of salt stress in Pokkali [22]. An incidence of high number of gene probes showing differential expression was also observed for Vandana (2479 gene probes for up- and 3074 for down-regulated) and IR64 (1710 gene probes up and 2192 down-regulated) in response to drought stress (Figure 1). Venn with all the four varieties and two stresses (salinity and drought) depicted 48 gene probes (27 genes) which were commonly differentially expressed in all the four varieties and two stresses. Those were stress inducible genes belong to heat shock proteins, DEAD helicases, zinc finger protein, glutamine synthatase (GST) and phospho ethanolamine. Microarray was employed to study cross talk of multiple stresses for cold, drought, high salinity, and/or abscisic acid (ABA), 15 genes were commonly expressed among all the four stresses which is dominated by genes related to signaling [23]. Our results indicate a common regulatory pathway for drought and salinity stress in tolerant and sensitive rice varieties [18, 23]. Genes always work in coordination and in the form of a network hence, co-expression gene interaction networks (GINs) were generated using differentially expressed genes (DEG).

### Co-expression GINs for DEG

Due to advent of high-throughput technologies we can formulate the biologically significant models to study the effect of environmental perturbations on cellular genetic and metabolic networks [24]. More advance bioinformatics analysis of microarray data can be judiciously employed to formulate the model for prediction of genes, which were co-expressed, and their correlations with abiotic stress conditions. Finally, we have attempted to create the models in predicting gene interaction networks by integrating computational and experimental approaches.

### GINs for genes common to salt stress in the tolerant and susceptible varieties

**Figure 2:**
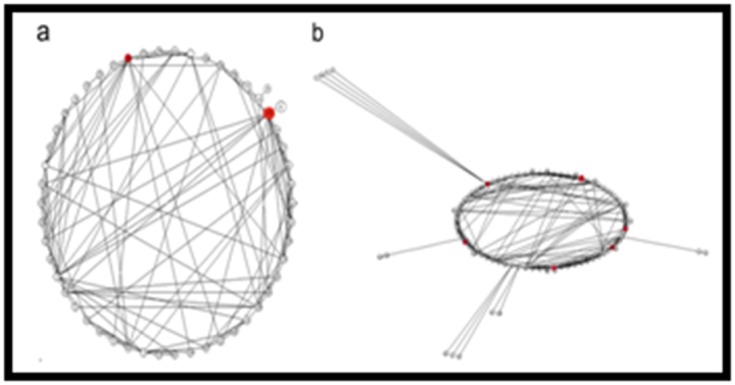
Gene interaction network for salinity stress induced differentially regulated genes in contrasting rice genotypes. Coexpression gene interaction network (A) for genes up regulated (B) down regulated in SS (VSR 156) and ST (CSR 11) during salinity stress as shown in Figure 1. Hubs are indicated with red circle and inferred interactions as edges.

A co-expressed GIN was formulated for the DEG of CSR11 and VSR156 under salt stress condition. A total of 23 genes (nodes) with 154 interactions (edges) were induced that formed a single GIN. The nodes were identified which showed maximum interactions to other nodes in the GIN and defined as 'hub'. Os.519.1.S1_at (LOC_Os01g08860, heat shock protein, Hsp18) that was connected to 14 gene probes which contain genes of secondary metabolic process, plasma membrane and molecular function (Figure 2a). Os.1000.1.S1_at (LOC_Os01g01660, isoflavone reductase) was connected to 14 gene probes including those for response to stress, plasma membrane, carbohydrate metabolic process, transferase activity, transporter activity, lipid metabolic process, lipid binding, isoflavone reductase homolog, import inner membrane translocase, metallothionein-like protein, 17.5 kDa class II heat shock protein, pathogen induced protein 2- 4, retrotransposon protein, abscisic stress ripening protein 2, conserved hypothetical protein and expressed proteins. Similarly GIN was also crafted for down regulated genes. Total 50 down regulated genes formed one co-expression GIN with 50 nodes and 230 edges. With Os.22353.3.S2_x_at (LOC_Os09g04210, hydrolase/zinc ion binding protein) as the hub gene connecting to 11 gene probes (Figure 2b) including, serine/threonine-protein kinase receptor, transcription regulator, electron transporter/ heat shock protein binding protein, RNA-binding protein containing a PIN domain, cytochrome b/b6/petB family protein, chloroplast 50S ribosomal protein, ribulose-1,5 bisphosphate carboxylase / oxygenase large subunit N-methyltransferase.

### Interaction network of genes for drought stress

#### GINs genes commonly up regulated under drought stress in IR64 and Vandana varieties

**Figure 3:**
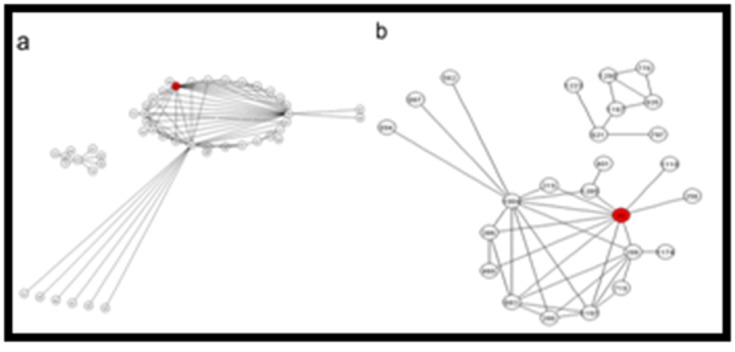
Gene interaction network for drought stress induced differentially regulated genes in contrasting rice genotypes. Coexpression gene interaction network (A) for genes up regulated (B) down regulated in DS (IR64) and DT (Vandana) during drought stress as shown in Figure 1. Hubs are indicated with red circle and inferred interactions as edges.

The GIN was represented by a small network of 25 nodes having two GINs (Figure 3a). Major GIN contains 18 nods with 39 edges and the smaller one with only 7 nodes having 29 edges. Os.11023.1.S2_a_at (LOC_Os01g51890, inositol-1, 4, 5- triphosphate) gene probe was connected to 10 gene probes which includes transcription factor activity and protein binding, desiccation-related protein, calcium-dependent protein kinase 2, glutathione S-transferase (GST), cytochrome P450, ATPdependent RNA helicase and F-box domain containing protein. GIN2 was containing kinase activity, catalytic activity, signal transduction transcription factor, transporter activity and hydrolase activity genes, protein kinase, calcium ion binding protein and expressed proteins.

#### GINs genes commonly down regulated under drought stress in IR64 and Vandana

This GIN contained 48 genes with two networks -GIN1 with 39 and GIN2 with 9 gene probes (Figure 3b). Os.26594.1.S1_at (LOC_Os07g40250, sex determination protein tasselseed-2) in GIN1 was connected to 14 number of genes which contain genes for signal transduction, transcription regulator activity, inositol-1, 4, 5-trisphosphate 5-Phosphatase, serine/threonine-protein kinase, and ABC transporter-like protein. GIN2 was very small network containing genes for signal transduction, and kinase activity.

#### GINs genes commonly regulated between drought and salt stress in all four varieties

**Figure 4:**
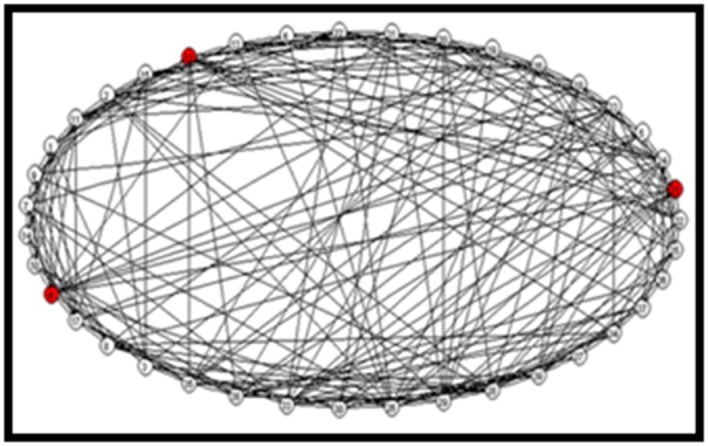
Gene interaction network for common genes, differentially expressed in salinity and drought stress in contrasting rice genotypes. Co-expression gene interaction network for genes up regulated or down regulated in SS (VSR156), DS (IR64), ST (CSR11) and DT (Vandana) during salt and drought stress as shown in Figure 1. Hubs are indicated with red circle and inferred interactions as edges.

We compared genes which were commonly differentially expressed under salinity and drought stress conditions because this leads to identification of stress responsive genes which may related to other stresses. A total of 39 genes were found to be common to both the stresses and a single dense Co-expression GIN was established (Figure 4). It has 3 major hubs (i) Os.14820.1.S2_s_at (LOC_Os05g51150, RNA polymerase sigma factor, transcription factor activity) connected to 14 nodes which included genes for transporter, response to biotic stimulus, amino acid and derivative metabolic process and kinase activity; (ii) OsAffx.17491.1.S1_at connected to 11 genes which included transporter activity, transferase activity, transcription factor activity, response to abiotic stimulus, kinase activity related genes. (iii) Os.37184.1.S1_at (LOC_Os01g43230, expressed protein) was also connected to 11 gene probes that included hydrolase activity, transferase activity, nucleic acid binding, protein metabolic process, response to abiotic stimulus and transport genes. A single co-expressed GIN was established for the genes induced and/or suppressed in salt tolerant and sensitive rice cultivars. Hub genes heat shock protein, Hsp18 (LOC_Os01g08860) and isoflavone reductase (LOC_Os01g01660) were found for up-regulated GIN. Similarly for down regulated GIN hydrolase/zinc ion binding protein (LOC_Os09g04210) gene was the hub gene. For drought treatments 2GINs were reported for induced and suppressed gene sets however the size of GIN was smaller than that of GINs for salinity treatment. The hub genes for up-regulated co-expression GIN were inositol-1, 4, 5- triphosphate (LOC_Os01g51890) and sex determination protein tasselseed-2 (LOC_Os07g40250) for down-regulated coexpression GIN.

A single and dens GIN was found for common co-expressed genes under both the stresses drought and salinity and 3 hubs were reported in the GIN. The GIN for salinity treatment for upregulated co-expressed genes was rich in heat shock protein, which was not only a hub gene but also a connecting gene. However, GIN for down-regulated co-expressed genes was enriched with units of kinases and signal transduction genes. Similarly GIN for drought treatment for induced co-expressed genes were enriched with transcription factors, kinases, helicases, GST, F-box proteins and signal transducers. GIN for commonly co-expressed genes was mixture of kinases, TFs and stress responses. Among all the co-expressed GINs kinases, TFs and Hsps were invariably over-represented indicating involvement of those gene families in abiotic stress tolerance and their conserved nature in all the varieties. Gene network were crafted for A. thaliana and high connectivity were observed for cellular function genes involved in response and adaptation to different environmental condition and conserved regulatory strategies were also detected [25]. Data set (15) of Affymetrix rice microarray were subjected to Weighted Gene Correlation Network Analysis (WGCNA), one of the network analysis tool and different gene modules were identified and two important annotation types were identified [26].

#### Identification of regulatory elements in salinity and drought stress

Cis-regulatory elements (CREs) present in the promoter region of genes interacted with TFs to induce the downstream genes, which lead to stress tolerance [27]. Therefore, CREs of genes, which were differentially expressed in salt and drought stress conditions individually and/or commonly, was analyzed. Those genes, which are retrieved from co-expression, GIN for salinity drought and common to both the stresses were picked and there promoter sequences were subjected to CREs analysis with motif search by employing MEME [21] and for motif validation tools. The predicted motif was searched against the sequence database for the occurrences using MAST (Motif-based sequenced analysis tool). MAST assumes exactly one occurrence of each motif per sequence, and each sequence in the database has assigned a pvalue, based on the product of the p-values of the individual motif occurrences in that sequence. The motifs predicted were compared and validated against the "JASPAR_CORE_2014_plants database" using TOMTOM tool. Total 64 motifs were read from this database after removing those, which has conflicting Ids. The significant matches were listed against each motif in the section "Significant motifs".

**Figure 5:**
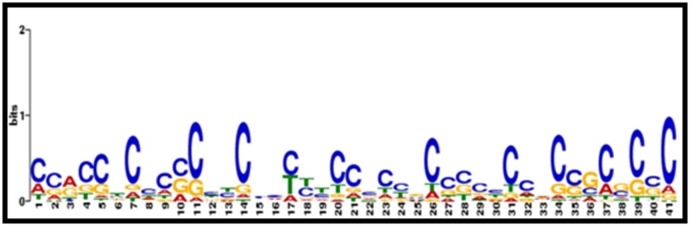
Over-representative cis-regulatory elements. Motif logos were generated for common genes, differentially expressed in salinity and drought stress in contrasting rice genotypes using MEME software. The overall height of each stack indicates the sequence conservation at that position (measured in bits), whereas the height of symbols within the stack reflects the relative frequencies of the corresponding nucleic acid at that position.

**Figure 6:**
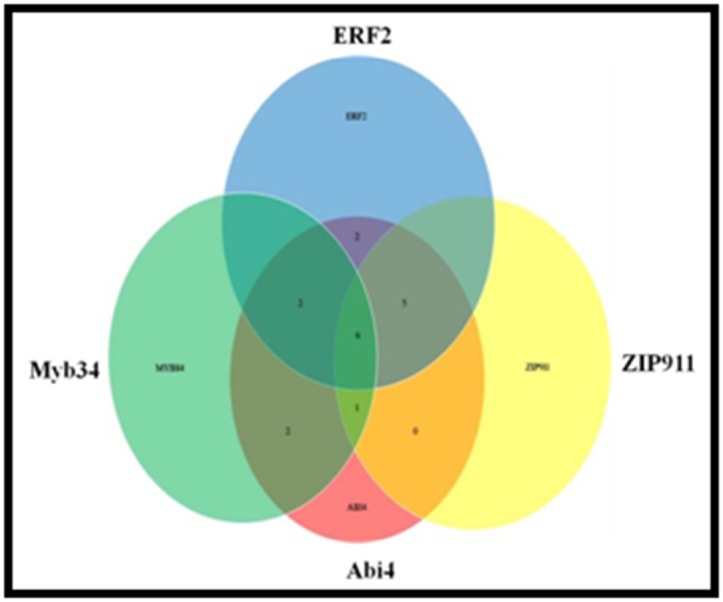
Venn for cis-elements for commonly differentially expressed co-expression GIN genes in salinity and drought stress conditions. Abi4, ERF2, Myb34 and ZIP911 transcription factors were over dominated in salinity and drought stress conditions in rice. Significant motifs (p≤0.05) present in the promoters of genes used for creating co-expression gene interaction network as shown in Figure 2, Figure 3, Figure 4 were mapped in rice genome.

We analyzed motifs for genes, which were commonly differentially expressed in salinity and drought stresses (Figure 5). For total 27 co-expressed genes, 20 significant motifs were detected (Figure 5). AgriGO analysis revealed the presence of TFs responsive to abiotic stresses. ABA-INSENSITIVE (Abi4) TF was over dominated for all the 20 motifs. Abi4 is an Apetala 2 (AP2)- type transcription factor responsive to various signaling pathways (ABA, sugar, ROS and salinity) in plant cell (Figure 6). Impaired drought tolerance was reported in mutant of abi4 [28]. In Arabidopsis Abi4 binds with the CE1 element [CACC(G)] in the promoter of its target genes that may be a core ABI4 binding elements in the genes suppresses by ABI4 [29]. After Abi4, 16 motifs for ERF2 were present followed by ZIP911 (12 motifs), Myb84 (10 motifs), Myb15 (7 motif) and Myb77 (5 motif). However, ERF were largely known for their role in biotic stress responses, some (AtERF1, AtERF2, AtERF3, AtERF4, and AtERF5) had been identified for abiotic stresses in Arabidopsis [30]. ERF1 was highly induced by salt and drought and various types of osmotic stresses in Arabidopsis. ERF1 induced specific sets of genes in response to salt and drought stress by stressexclusive binding to GCC [7, 30]. For the first time in this study ERF2 was overrepresented with response to salt and drought stress of rice showing its significance in both the stresses. In rice there were 89 ZIPS were reported among them 37 were found to be differentially expressed in dehydration, salinity and cold stress [31] but there was no report was available for ZIP911 which is first time reported in this study. Rice R2R3-type MYB gene, MYB2 played regulatory role in tolerance of rice to salt, cold, and dehydration stress [32]. Conclusively, CACC and GCC, CREs were major performers for salt and drought stress responsive mechanism. However, detailed analysis to confirm the role of these TFs and cis-regulatory elements has warranted for future study.

#### Validation of commonly regulated genes for differential expression using RT-PCR

Among 27 common DEG, 10 genes on the basis of their expression value in microarray were selected for their quantitative expression in leaf tissues of CSR11, VSR156 salt stressed and without stress, similarly for drought responses Vandana and IR64 stressed and without stressed were used. RNA was extracted and cDNA was synthesized using Superscript III. Real time PCR was performed with 10 genes using alpha elongation factor (α-Efa) as housekeeping genes for internal control. LOC_Os03g563700 (molecular function, Omethyltransferase) was induced under salt stressed conditions while being strongly suppressed in drought stress in both sensitive and tolerant genotypes. LOC_Os12g19530.1 (nucleotide binding, ATP-binding region ATPase like protein) was down regulated in both stresses for all the four genotypes. LOC_08g07540 (response to stress, hemimythylated DNAbinding protein) showed up-regulation under salt stress for CSR11 while, negligible changes was reported for VSR156. However, under drought stress, Vandana showed down regulation and a marginal change for IR64 was observed. LOC_Os05g40270 (response to stress, ATP binding protein) was induced in salt stress conditions for both tolerant and sensitive genotypes; however, its expression was 10 fold higher in sensitive as compared to the tolerant variety. Similarly, gene LOC_Os05g40270 (response to stress, ATP binding protein) was down regulated in drought stress in Vandana while it was up regulated in IR64. LOC_Os09g24530 (ribulose-1, 5 bisphosphate carboxylase/oxygenase large subunit N-methyltransferase) was suppressed under both stress conditions but was more strongly pronounced in the sensitive genotypes as VSR156 an increase in down regulation by 1.5 fold and IR64 was exhibited a 1.4 fold drop in down regulation in comparison to tolerant genotypes. Os04g011740 (heat shock protein) was up regulated in salt and drought and was 17-fold higher in VSR156 in comparison to CSR11 for salt stress; it was 1.7 fold higher in IR64 as compared to Vandana for drought stress. LOC_Os06g045710 (phospho glycerate kinase) was upregulated under salt stress while under drought stress marginal change was observed. LOC_Os07g048870 (MYB family TF) showed a 10-fold increase induction under salt stress in VSR156 whereas a 5-fold induction in IR64 was noted under drought stress conditions. LOC_Os01g08860 (hsp20/ alpha crystalline family) was induced under salt stress and was 2.6 fold higher in VSR156 as compared to CSR11 and down regulated under drought stress and this suppression was 5-fold higher in Vandana as compared to IR64. LOC_Os03g016920 (DnaK family protein) was up regulated under both the stresses and it was 5-fold higher in VSR156 for salt stress and 4-fold higher in IR64 under drought stress as compared to control conditions. It can be concluded that suppression of O-methyltransferase (Os03g0775000), hemimythylated DNA-binding protein (Os08g0172200, LOC_Os05g40270) ATP binding protein and hsp20/ alpha crystalline family (Os01g0184100) genes have significant effect on drought tolerance. However, induction of hemimethylated DNAbinding protein (Os08g0172200), phosphoglycerate kinase (Os06g0668200), and 17.5 kDa heat shock protein (Os01g0184100) genes were important for coping salinity stress tolerance. Moreover, higher expression of MYB TF in sensitive genotypes depicted its significant function for salt and drought tolerance.

Genes which are commonly expressed in salinity and drought stress were ATP binding protein, ribulose-1, 5 bisphosphate carboxylase/oxygenase large subunit N-methyltransferase, 17.5 kDa class II heat shock protein, heat shock cognate 70 kDa protein which has also been discussed earlier for their role in drought and salinity stress. We validated 10 genes among 29, which are commonly expressed through qRT-PCR. Enzymatic methylation catalyzed by O-methyltransferases is one of the most important reactions in the complex phenylpropanoids and flavonoid metabolism. Caffeoyl CoA and caffeic acid OMT are able to methylate lignin precursors. The wheat TaoMTi gene responded to stress and its transcripts showed significant accumulation in leaves and roots treated by MeJA, ethylene, ABA, wounding, PEG and UV-B. An RNAi line of CcoAOMT was developed and wilting was found phenotypically in the transgenic under water stress. COMT RNAi lines showed more than 30% reduction in cell viability and wilting in leaves during high sunshine hours may be due to possible reduction of lignin leaves. Arabidopsis mutants were susceptible to salinity, water deficit stress and disease resistance [33]. In our study, it is up regulated in salt stress but down regulated in drought stress conditions. O-methyltransferase is important for salinity tolerance as compared to drought. Plants are exposed to stress conditions during salinity stress, namely ionic and osmotic stress while under the drought situation, only osmotic stress persists. An increased lignin synthesis is required to tolerate salt stress. Hemi-methylated DNA binding proteins are induced under salinity stress in tolerant lines while being down regulated in drought stress conditions. This also showed the importance of methylation reactions in salt stress more than drought stress. ATP is the primary source of energy for plants and under stress conditions plants require more energy to survive under stress. Sensitive lines of salt and drought showed up-regulation of ATP binding protein while tolerant ones were down regulated. Metabolism of sensitive plants was much more disturbed then tolerant ones owing to lower stress tolerance and subsequently requires more energy derived metabolically for their survival. OsMYB2, a R2-R3 type MYB gene played pivotal role in multiple stress tolerance of rice [32]. OsMYB2 over expressing rice could accumulate higher osmolytes as soluble sugars, proline and LEA proteins and reduce MDA and H2O2 under salinity stress condition, thus protect plant by regulating osmotic balance and oxidative damage [32].

## Conclusions

It is of interest to explore the interaction of genes associated with drought and salt stress responses in tolerant and sensitive varieties of rice to help identify cis-regulatory elements responsible for regulation of genes in both the conditions. Novel insights into condition-specific gene interaction and cisregulatory elements were obtained which demonstrates the strength of the GIN approach to infer gene networks

## Competing Interests

We declare no competing interest with any one.

## Author’s contributions

PM and VM and PG carried out the experiments, NS helped in bioinformatics analysis and contributed in writing, SPK helped in bioinformatics analysis, AJ contributed in writing, VR and NKS conceptualized the idea and designed the whole work.
